# Supplementary material for: Cardio PyMEA: A user-friendly, open-source Python application for cardiomyocyte microelectrode array analysis
Source: PLoS One. 2022 May 26;17(5):e0266647. doi: 10.1371/journal.pone.0266647 (PMC9135279; doi:10.1371/journal.pone.0266647)
Supplement: Attachment — Submitted filename: review_PONE-D-22-08656.pdf [file pone.0266647.s004.pdf]

Review for PLOS ONE

Manuscript: Cardio PyMEA: A user-friendly, open-source Python application for cardiomyocyte microelectrode array analysis

Manuscript ID: PONE-D-22-08656

April 12, 2022

The present work presents an open-source Python graphical application for the study of microelectrode array analysis (MEA) of cardiomyocytes. The application offers a series of fundamental features for loading, analyzing, and exporting the data from MEA recordings. A series of calculations such as beat detection, electrode exclusion, electrode silencing, pacemaker origin detection, local activation time, maximum upstroke velocity, beat amplitude and interval, conduction velocity, pacemaker translocation detection, and statistical analyses are available. The manuscript is very well written, well organized, relevant, and the presented results are encouraging and significant. The reviewer has no major or general comments, since the manuscript and the contribution are already very clear and significant. However a list of suggestions for improving the text of the manuscript and for future upgrades on the software are provided below, together with some specific or minor comments.

Suggestions for improving the manuscript

1. Sample data for immediate usage and test of PyMEA could be made available in the Github repository.

2. The abstract is very focused on the software license and other computational aspects of PyMEA. However, the reviewer believes that providing a compact and general overview of the main capabilities of the software (beat detection, pacemaker origin estimation, and etc) on the abstract could further gain the attention of researchers working within this field and looking for this kind of solution.

3. The authors could explain the method used to compute the field potential duration (FPD) according to the mentioned reference using a simple and compact approach. All the other details from the features of the software are well explained, with the exception of this calculation.

Specific comments and suggestions

1. No page or line numbering are provided in the manuscript. This makes the process of reviewing a little bit harder.

2. Section "Automated electrode exclusion". Instead of using the term *data modification*, the reviewer thinks that *data treatment* or *data pre-processing* is more adequate.

3. After the equation of the radius r, it is said: *These parameters are additionally bound.* It seems the authors are referring to imposing bounds on the parameters during the least-squares fitting. Please make it clearer.

4. LAT was defined 2 times or more.

5. Please specify what type of finite difference was applied to approximate the derivatives for the maximum upstroke velocity calculation (forward,backward,central,...).

6. Conduction velocity calculation. Although very simple and easy to infer, the *formula* "electrode-distance/ΔLAT", lacks the definition of ΔLAT.

7. Property vs distance from pacemaker. Please define *multiplier*

8. In general the texts describing the name of functions within the code are written with another font style. For LaTeX users this is usually done with the command `texttt`.

9. Please explain or provide a reference for Sturge's rule.

10. It seems the concepts of power-law and probability density functions are mixed or entwined. he authors

11. Should some signal processing tools be used for noisy signals before MEA calculations? Is this a common practice within the field? Perhaps the authors could mention something about this.

12. Cardio PyMEA provides uniques (section). Before the caption of Fig.8 the authors mention "*suggesting that there is a strong relationship between these two properties*", when refering to the R^2 coefficient of determination. Perhaps using "*suggesting a good fitting was achieved*", or something similar would be better, to avoid a possible confusion with a positive correlation.

Suggestions for future improvements of the software

1. At the final section of the manuscript the authors mention the software may freeze when using large datasets. On the reviewer's experience this may be related to the usage of the `matplotlib` as a backend for the plots. Although it is pretty standard for

Python code to use `matplotlib`, for huge datasets it takes significant time to render and plot data with this library. An alternative is to use the `PyQtGraph` library (https://www.pyqtgraph.org/), which is faster and can easily handle large datasets. It can also deliver high-quality plots.

2. Currently PyMEA only supports input data in the format of a specific MEA recording software. For a broader use, this could be extended to other input formats which might be also in use within the MEA community.

3. Also with respect to input/output, the authors could consider in the future using data in the HDF5 file format. It can significantly reduce storage of large datasets, and may provide a faster interface for reading and writing data, which could also impact on the performance of the software when loading large datasets.

4. Please check the `OpenCARP` suite for cardiac electrophysiology, which provides the `limpetgui`, a tool for visualization of large datasets of cardiac action potential models. It uses both `PyQtGraph` and HDF5.
   Link: https://opencarp.org/documentation/examples/visualization/limpetgui

5. Another interesting feature for the future, would be the possibility of loading two datasets for comparisons and computation of quantitive metrics about their differences, in case this could be useful for MEA studies.
